# Supplementary material for: The impact of VUS reclassification on reproductive decision making
Source: PLoS One. 2025 May 27;20(5):e0308771. doi: 10.1371/journal.pone.0308771 (PMC12111534; doi:10.1371/journal.pone.0308771)
Supplement: Attachment — Submitted filename: Preconception VUS.pdf [file pone.0308771.s002.pdf]

Variant discovery resulting in VUS during preconception recessive and X-linked carrier testing is an important issue in the medical literature. Defining precisely what the authors consider to be carrier screening would benefit this paper as there are a few areas in which the definitions cross boundaries into other types of genetic testing in the prenatal setting. Prenatal genetic testing differs across jurisdictions – the paper would benefit if the authors state what specific issue within their jurisdiction needs to be resolved. Please find some comments on the proposed paper:

Abstract

"Laboratories will occasionally upgrade a VUS to pathogenic (P) or likely pathogenic (LP), making it clinically actionable."

- Variant reclassification may result in the changed classification of any variant to any other distinct category and is not restricted to an "upgrade"

Introduction

There are three distinct entities within genetic testing in the prenatal setting that seem to be viewed interchangeably by the authors. The aim of the authors study appears to be point 1 below, and this study conflates an overlap between 2 & 3:

1. Asymptomatic recessive and X-linked carrier testing within prenatal medicine which is indicated for an unaffected (asymptomatic) individual without any unprobed family history of a genetic condition
2. Non-invasive prenatal testing using chromosomal microarray technology
3. VUS that are uncovered during the course of diagnostic testing in an affected individual, thereby leaving the case unsolved and no option for prenatal/preconception genetic testing for a **known** condition available to closely related family members. (Clinically, risk could be established for an unaffected but closely related couple using carrier frequencies to estimate risks of a rare recessive condition).

e.g. "Patients with a family history of a heritable disease with a VUS in a gene associated with that disorder present a unique challenge to the reproductive geneticist".

- The proper steps for a genetic diagnosis in a family for which preconception genetic testing can be offered is to make a diagnosis in the affected individual. The focus of the paper appears to be on asymptomatic recessive and X-linked carrier testing in prenatal medicine which is performed independent of a diagnosis in consideration. Therefore this statement appears to confuse diagnostic versus carrier testing. (Screening for autosomal recessive and X-linked conditions during pregnancy and preconception: a practice resource of the American College of Medical Genetics and Genomics (ACMG))

"VUS's are generally considered not actionable. Indeed, most PGT labs do not test embryos for a VUS" –

- VUS are always considered not actionable at the point of classification. VUS testing of an embryo may occur in select instances and is not really generalisable to the point of

this paper as a whole (Laboratory testing for preconception/prenatal carrier screening: A technical standard of the American College of Medical Genetics and Genomics (ACMG))

“VUS reporting during the pre-conception period presents a significant challenge to reproductive specialists. When a VUS is reported, the question arises whether pre-implantation genetic testing for monogenic disease (PGT-M) of embryos should be recommended to prevent the offspring from inheriting the variant.”

- The authors should emphasise that risk for the genetic variants classified as VUS during prenatal carrier testing is only significant in light of their partner’s status. Therefore, this is a leap to say that a VUS in a recessive gene may have clinical implications when you don’t consider the partner’s risk.

“the common reporting of VUS presents a difficult dilemma”

- Do the authors have a reference for how commonly VUS are reported in the prenatal setting?

“The ongoing reclassification of VUS, coupled with conflicting reporting by testing laboratories of the pathogenic significance of variants has led to confusion regarding the need for preconception testing of embryos for the variant in question (3).”

- The authors repeatedly reference laboratory reporting of discordant classification of the same variant. This is a separate issue to VUS reclassification and although it is examined in the methods of this study, may not contribute to ultimate genetic counselling. Can the authors please define the significance of variant discordance within the context of the study aims?
- “We did not report on those VUS’s in conflict with a benign and likely benign classification as our focus was on those VUS that are actionable leading to potential use of PGT-M.” – I note this as the final statement of the Discussion which provides useful information about the aim of the paper overall and would be important in the Introduction to clarify the aims.

“There is a dual dilemma; first, how should the patient be counselled when the variant is reported as VUS by one or more labs and as P or LP by another lab(s)”

- Genetic counselling is clinically confined to the genetic test report and the consideration of the testing centre and clinician interpretation.

“The second dilemma is how to relate to a VUS reported, for instance, for a cancer gene, where there is a significant familial history of the cancer associated with the gene (e.g., breast/ovarian cancer and BRCA1/2; colon cancer and APC).”

- This would require clarification as the authors previously state that the ACMG recommended gene panel for preconception genetic testing is the foundation of this study. However, cancer associated genes are not included in this panel. Can the authors please clarify this?

Materials and methods:

“The top 10 genes with the highest number of VUS’s per gene as well as the highest percent in conflict (defined as a VUS in conflict divided by the total number of VUS) were compared over the 3 years.”

- Why did the authors restrict their analysis to 10 genes?
- Why did the authors confine the time period to 3 years of data?
- Discordant submissions in ClinVar are not the same as VUS classification of variants and the authors should note the distinction

Discussion:

“VUS disclosure has the potential to increase parental distress as well create difficulties for genetic counselors as to how to advise patients (15). I”

- This distress seems to arise from the disclosure of CNV VUS detected during NIPT for an active pregnancy. Do the authors feel that this may extend to preconception counselling for what may be non clinically relevant VUS in recessive genes?

“. Our disease (cancer included) prevention mission is handicapped by the currently inefficient setting”

- Again, can the authors please clarify what preconception carrier testing methods report pathogenic or likely pathogenic variants in cancer related genes?

“Reporting to ClinVar is not mandatory”.

- However ClinVar still represents a rich repository of human genetic variation with over 4 million genetic variants submitted. It has proven use in the literature as an adjunct to variant classification and therefore does not represent a limitation of variant classification or reclassification.

“We may assume that given the exponential increase in VUS and VUS-in-conflict reporting, these numbers are only going to increase several-fold within 5,10 and 20 years.”

- Standardised guidelines and improved sequencing and bioinformatic techniques are resolving unsolved variation within the human genome. Do the authors have a reference to support this statement? Otherwise, it is in conflict with the current published literature.

“What will happen if VUS’s in conflict reach 25% of all cases?”

- Can the authors clarify the implications of this statement? What would happen? And is it likely to happen?

“Currently, many genetic labs do not report all VUS’s and therefore, the true incidence of potentially upgradable VUS’s is difficult to determine but likely, involves many more under-reported cases.”

- The study methods use ClinVar miner which is taken from ClinVar. ClinVar is a variant submission database, not a genetic test report database. Therefore, submitting labs and facilities submit any variant uncovered during genetic testing, and not just those that are clinically relevant for the test performed. Therefore this statement is inaccurate as VUS within ClinVar are more likely to closely represent all VUS detected during genetic diagnostic testing and research settings and not just those that reach the eyes of the clinician and patient on the final genetic test report.
